# Supplementary material for: On demand expression control of endogenous genes with DExCon, DExogron and LUXon reveals differential dynamics of Rab11 family members
Source: eLife. 2022 Jun 16;11:e76651. doi: 10.7554/eLife.76651 (PMC9203059; doi:10.7554/eLife.76651)

DExCon, DExogron, LUXon: on-demand expression control of endogenous genes reveals differential dynamics of Rab11 family members

Jakub Gemperle[1*], Thomas Harrison[1], Chloe Flett[1], Antony Adamson[1], Patrick Caswell[1+*]

[1]Wellcome Trust Centre for Cell-Matrix Research, School of Biological Sciences, Faculty of Biology Medicine and Health, Manchester Academic Health Science Centre, The University of Manchester, Manchester, UK.

*Correspondence: patrick.caswell@manchester.ac.uk; jakub.gemperle@gmail.com

+Lead Contact

UNCROPPED BLOTS

Figure 1- source data 1

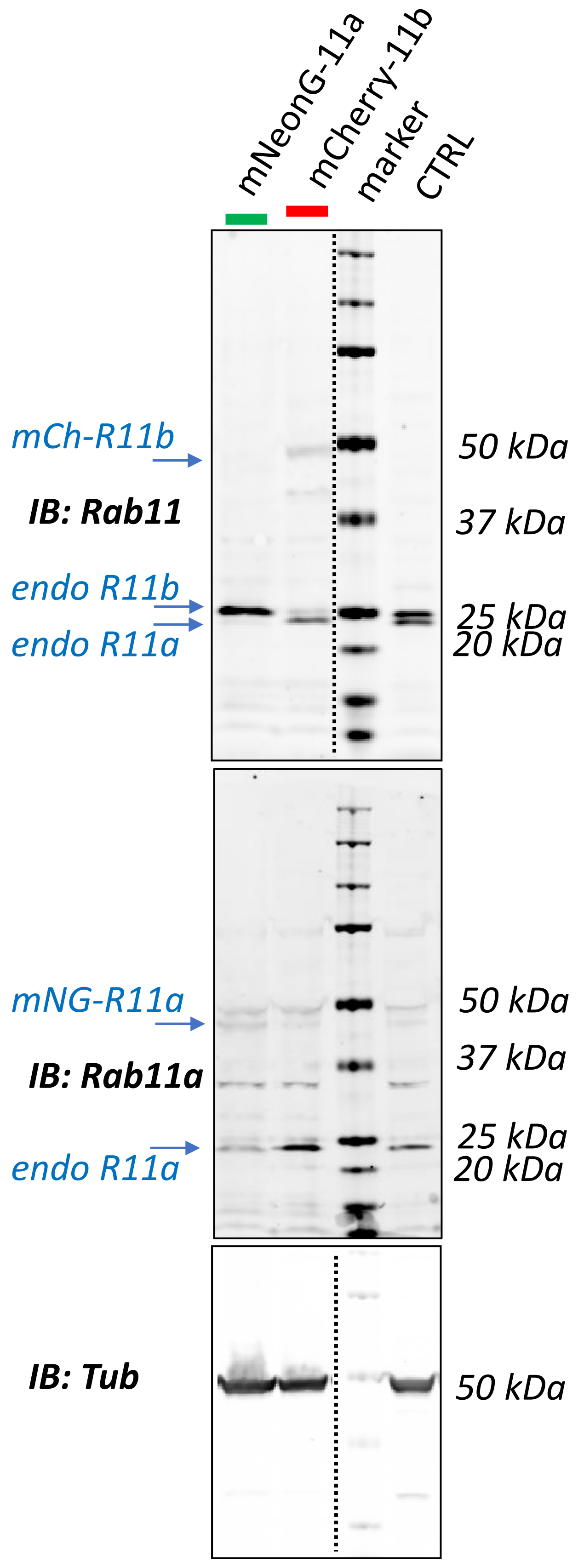

Figure 1-figure supplement 2 - source data 1

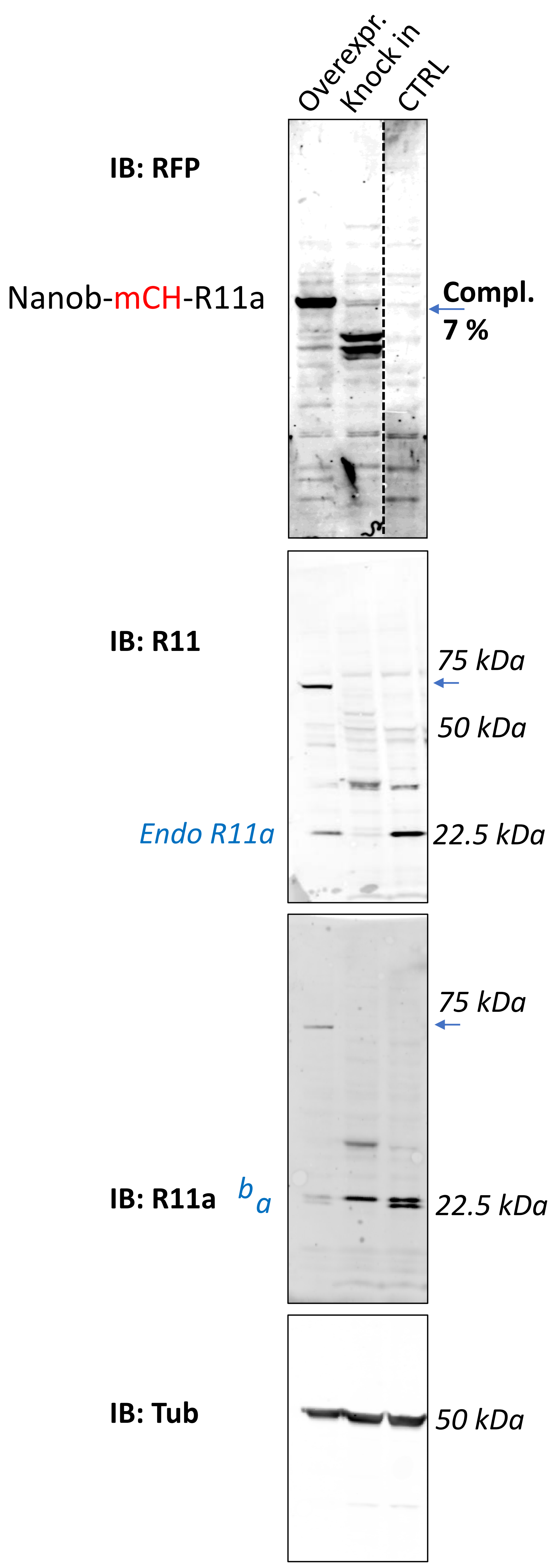

Figure 2- source data 1

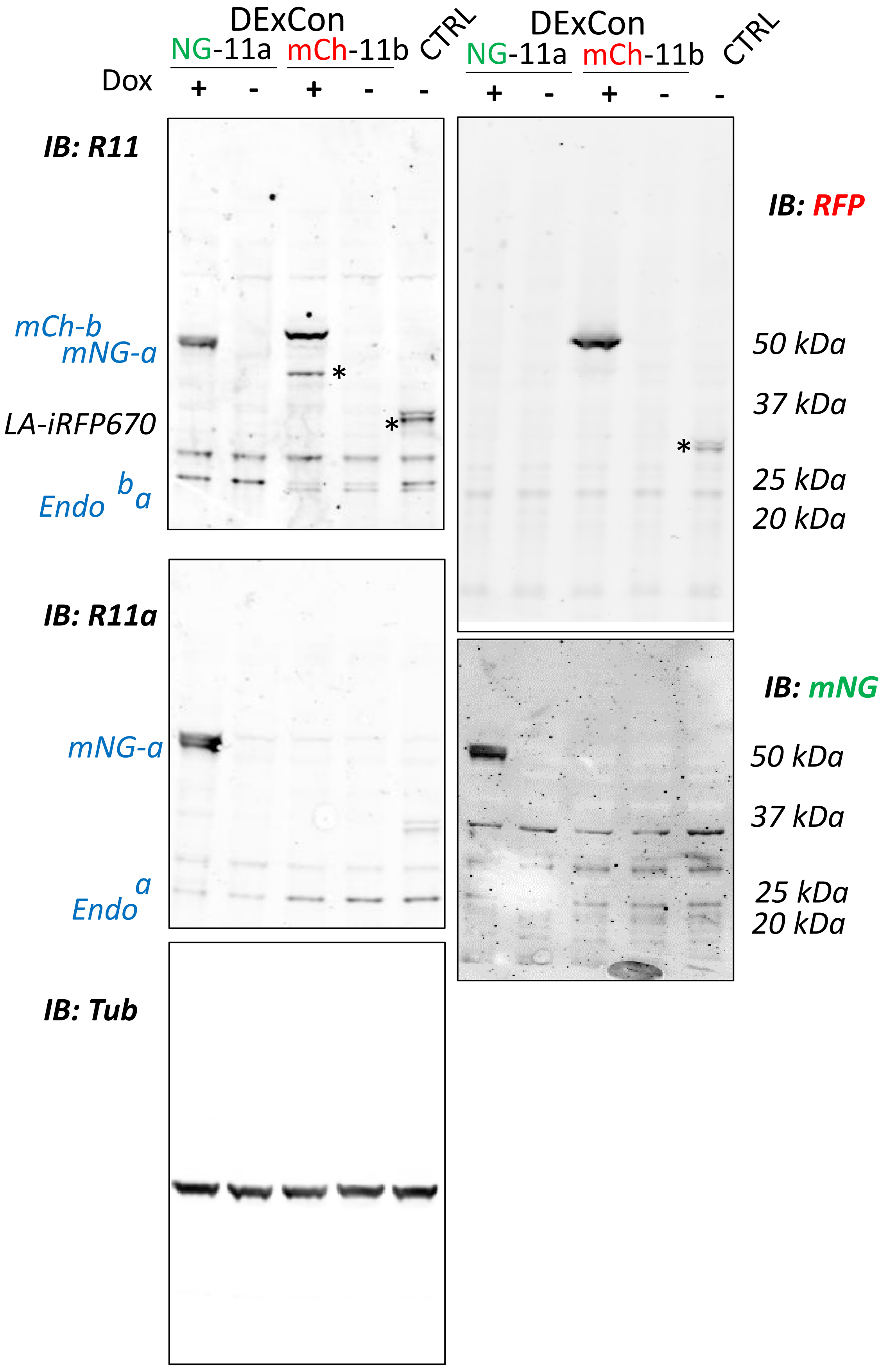

Figure 2- source data 2

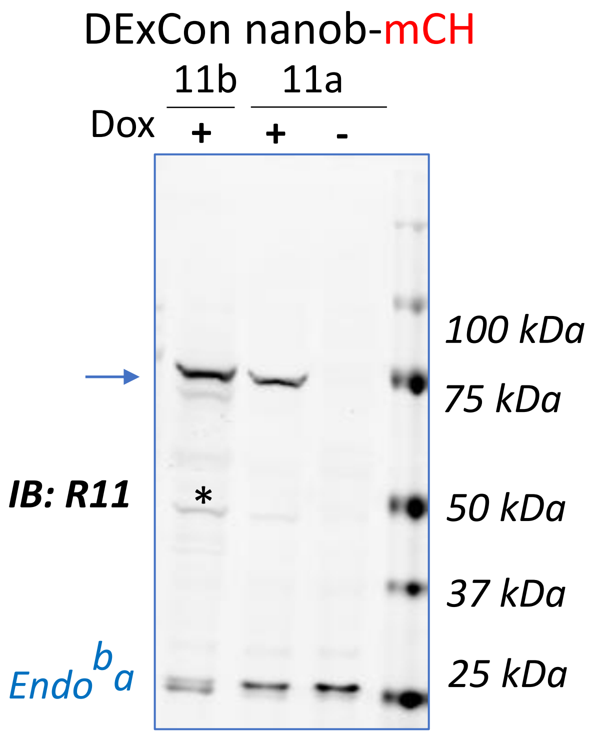

Figure 2-figure supplement 1 - source data 1

Source data same as for Figure 2 – source data 1

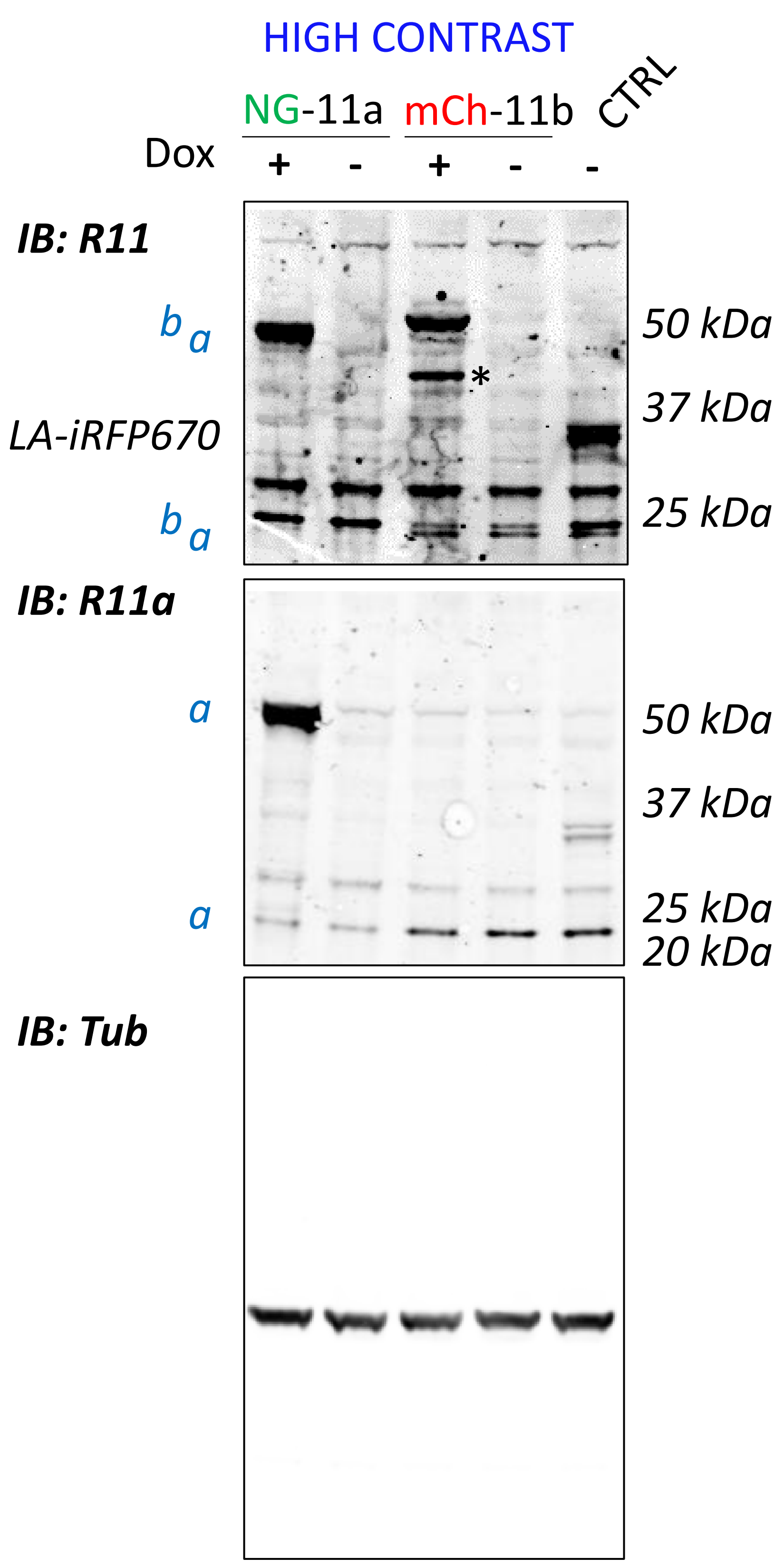

Figure 3- source data 1

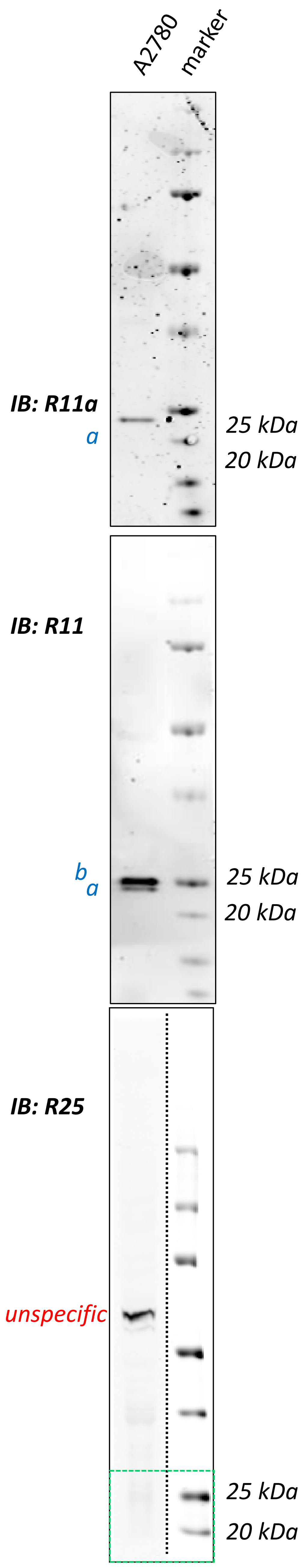

Figure 3- source data 2

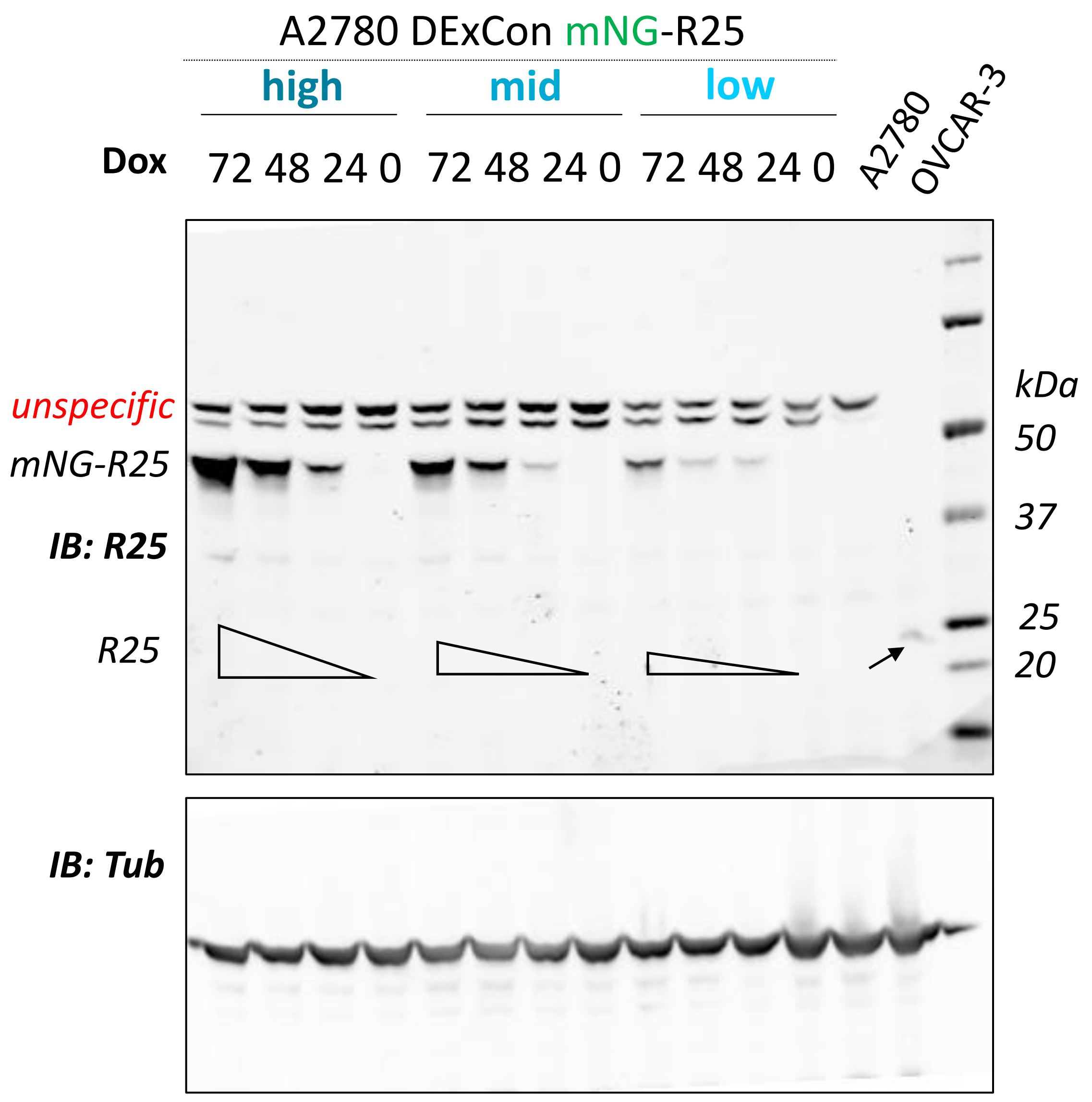

Figure 3-figure supplement 1 - source data 1

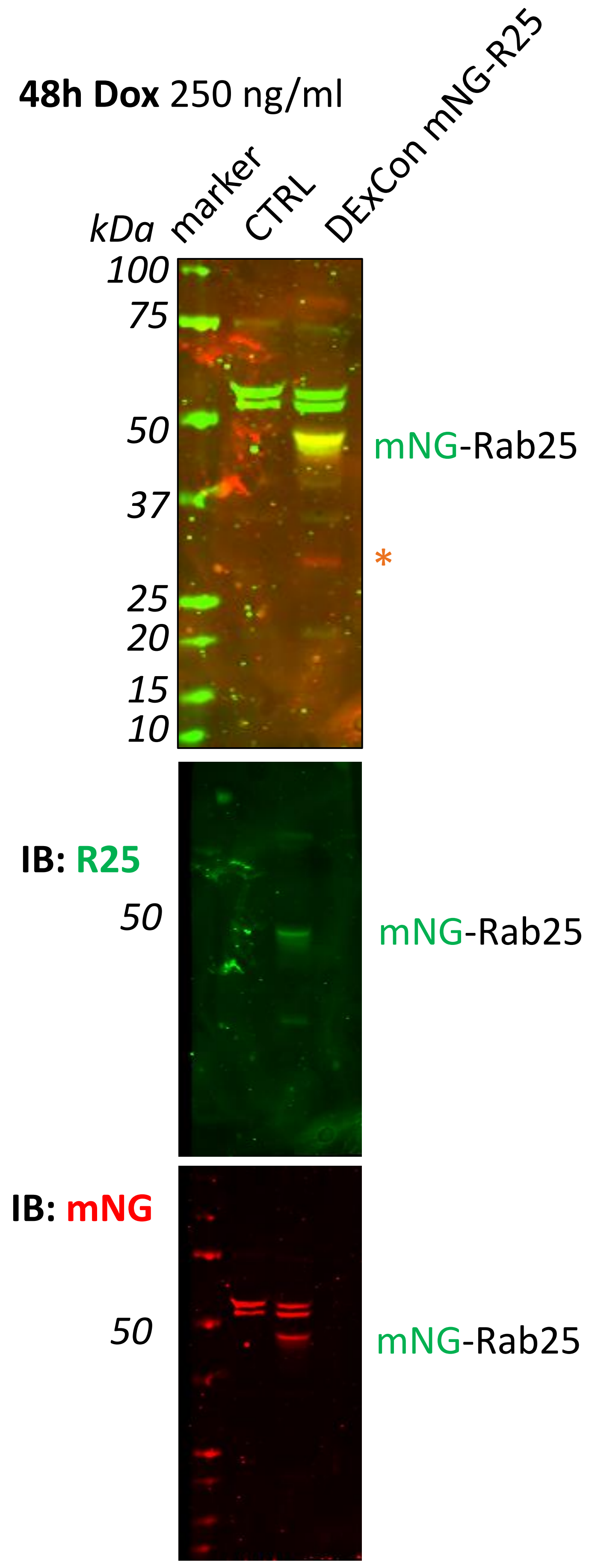

Figure 3-figure supplement 1 - source data 2

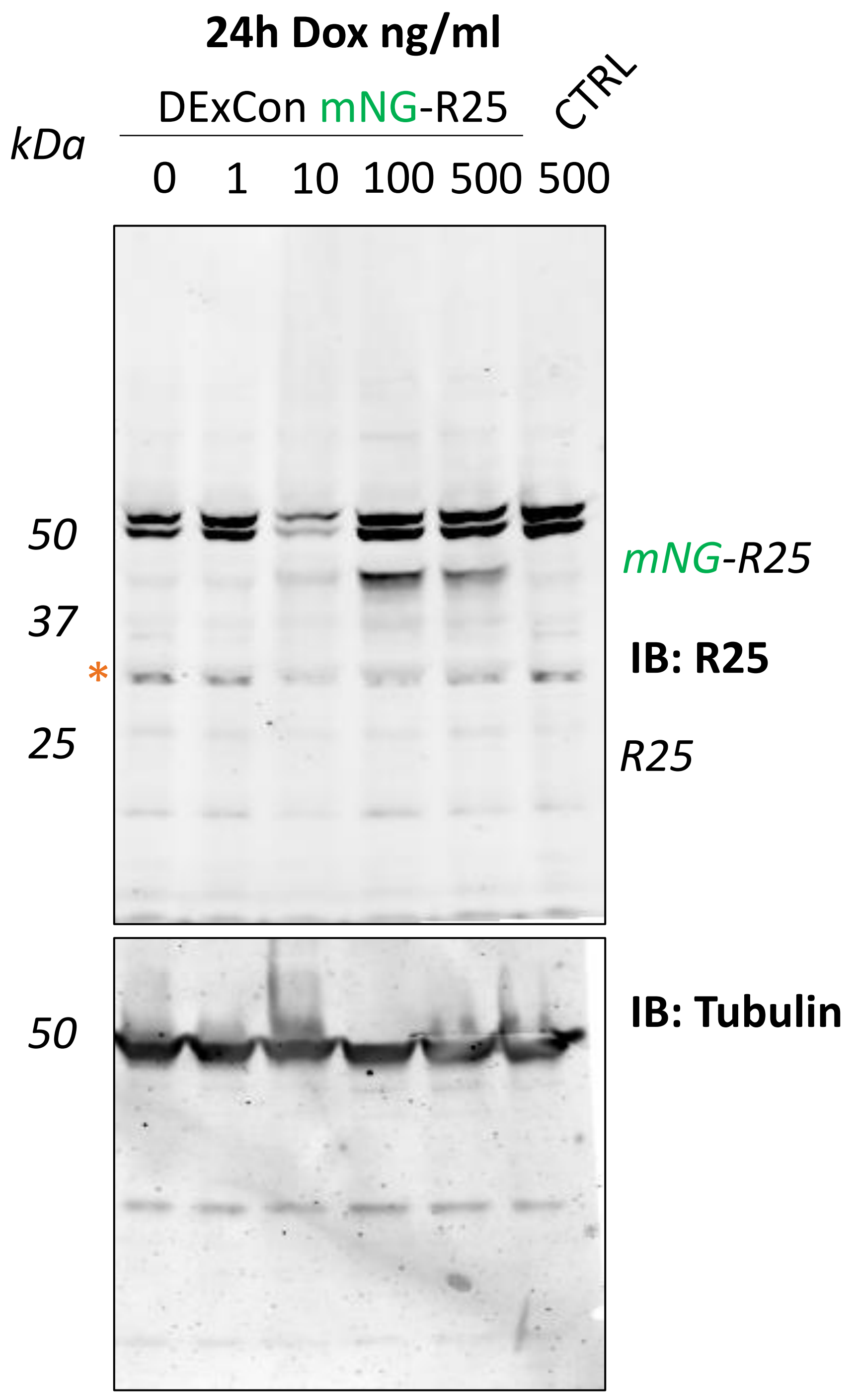

Figure 4-figure supplement 1 - source data 1

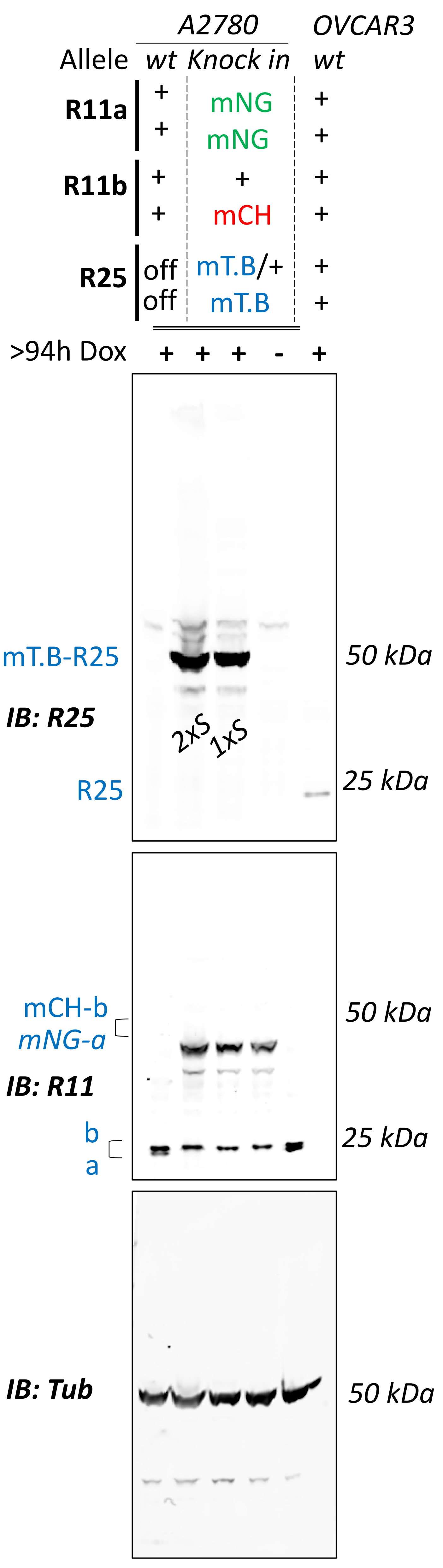

Figure 5- source data 1

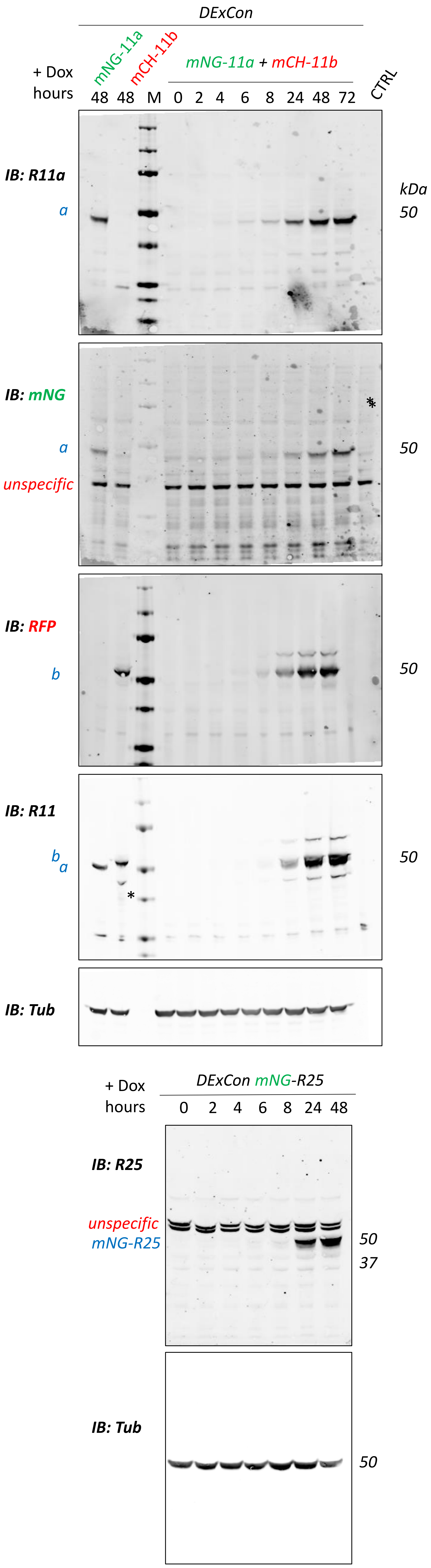

Figure 5- source data 2

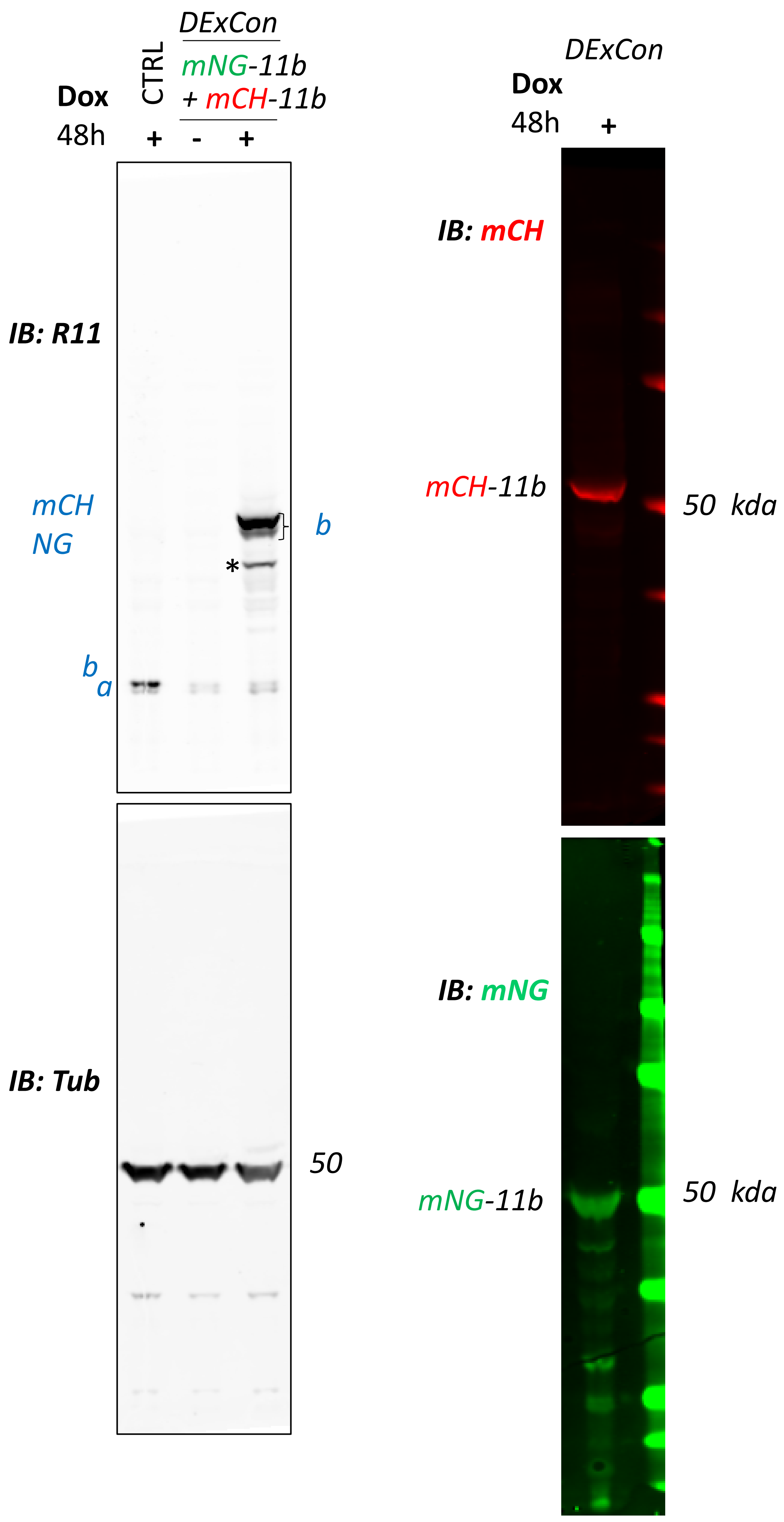

Figure 6- source data 1

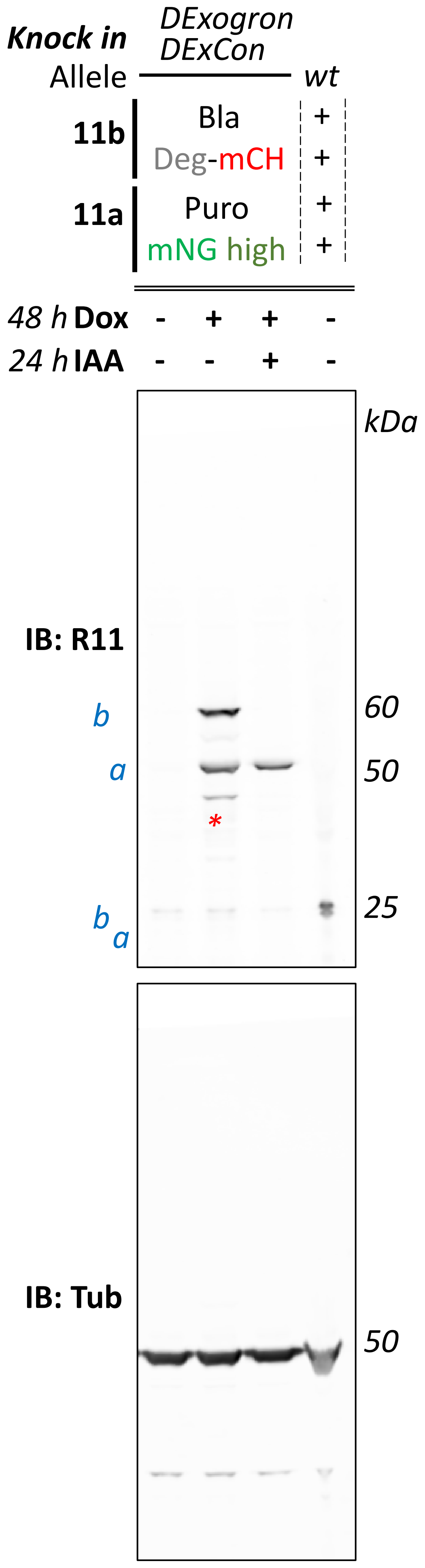

Figure 6-figure supplement 1 - source data 1

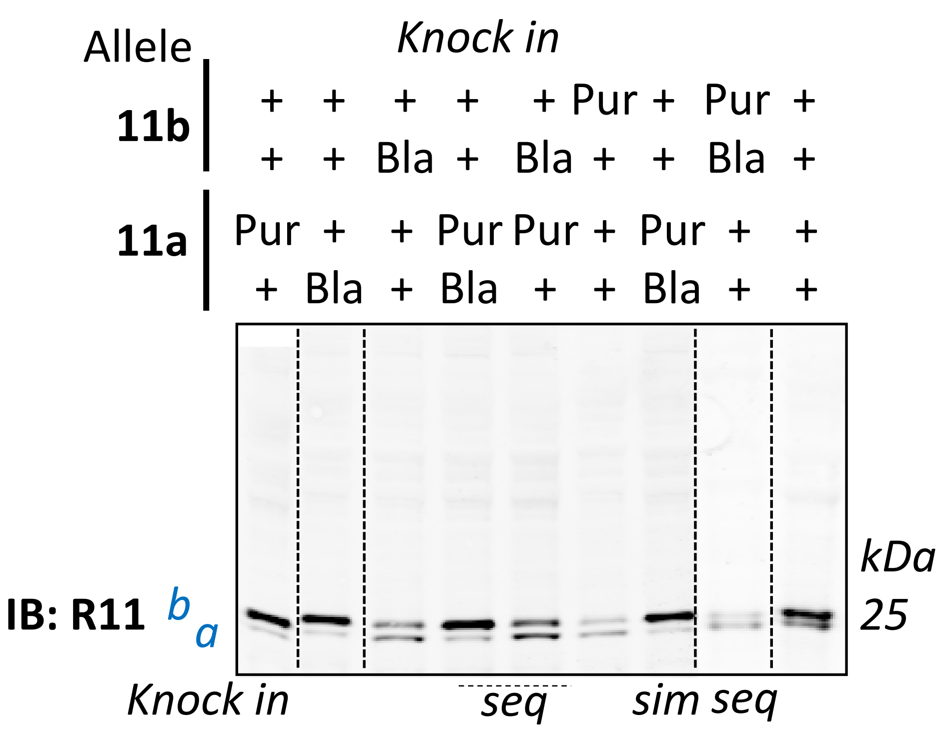

Figure 6-figure supplement 1 - source data 2

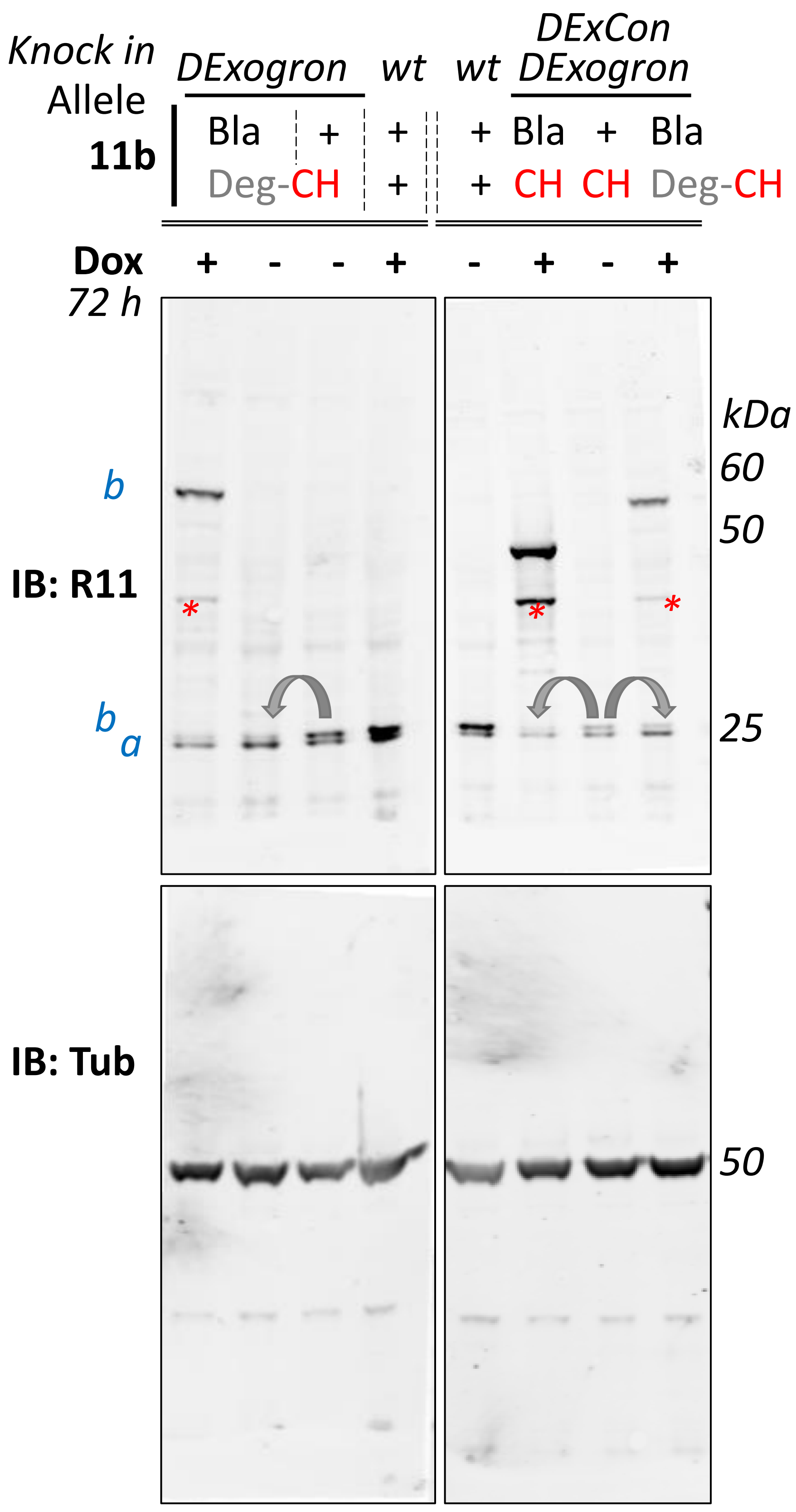

Figure 6-figure supplement 1 - source data 3

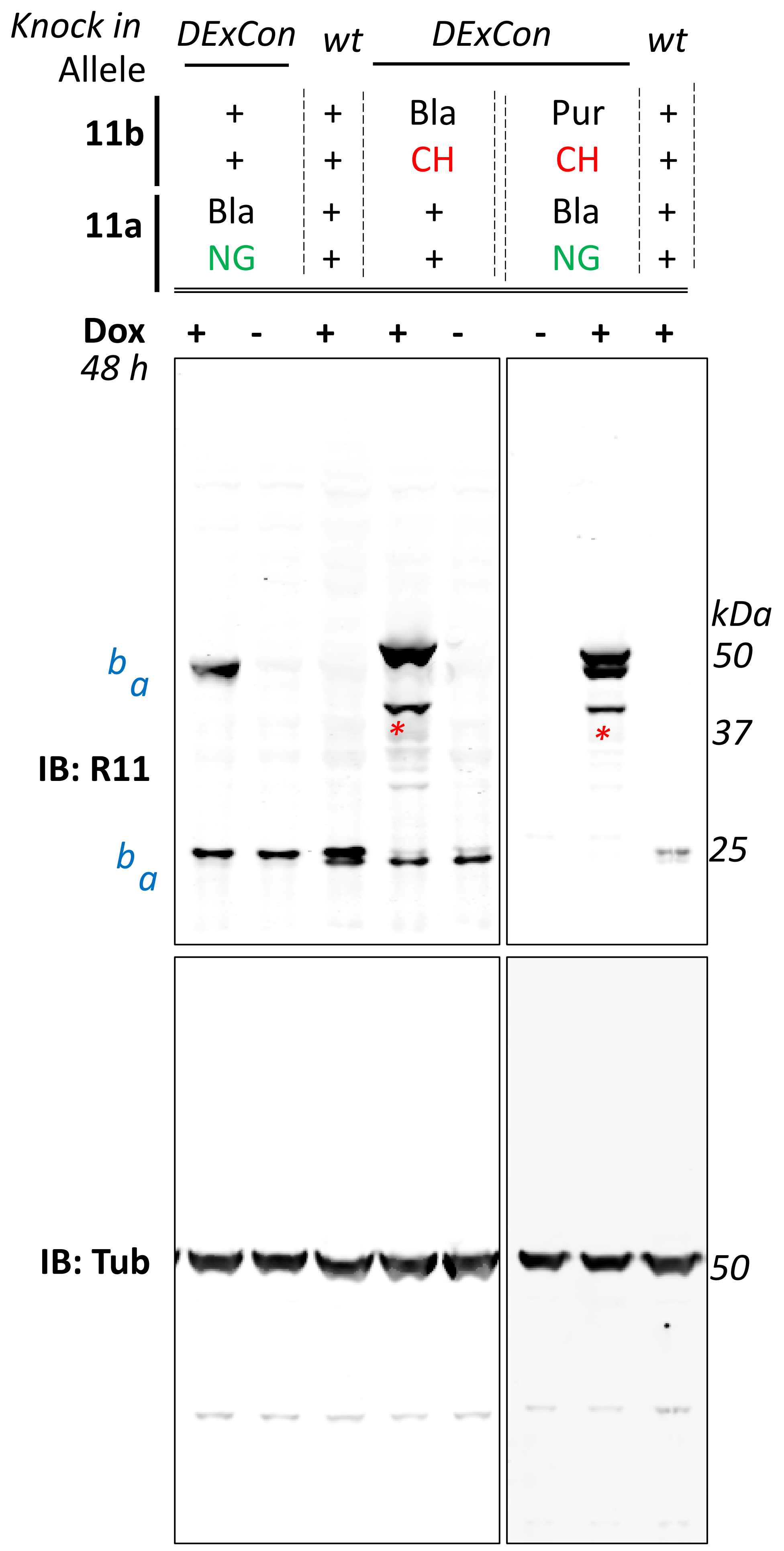

Figure 6-figure supplement 1 - source data 4

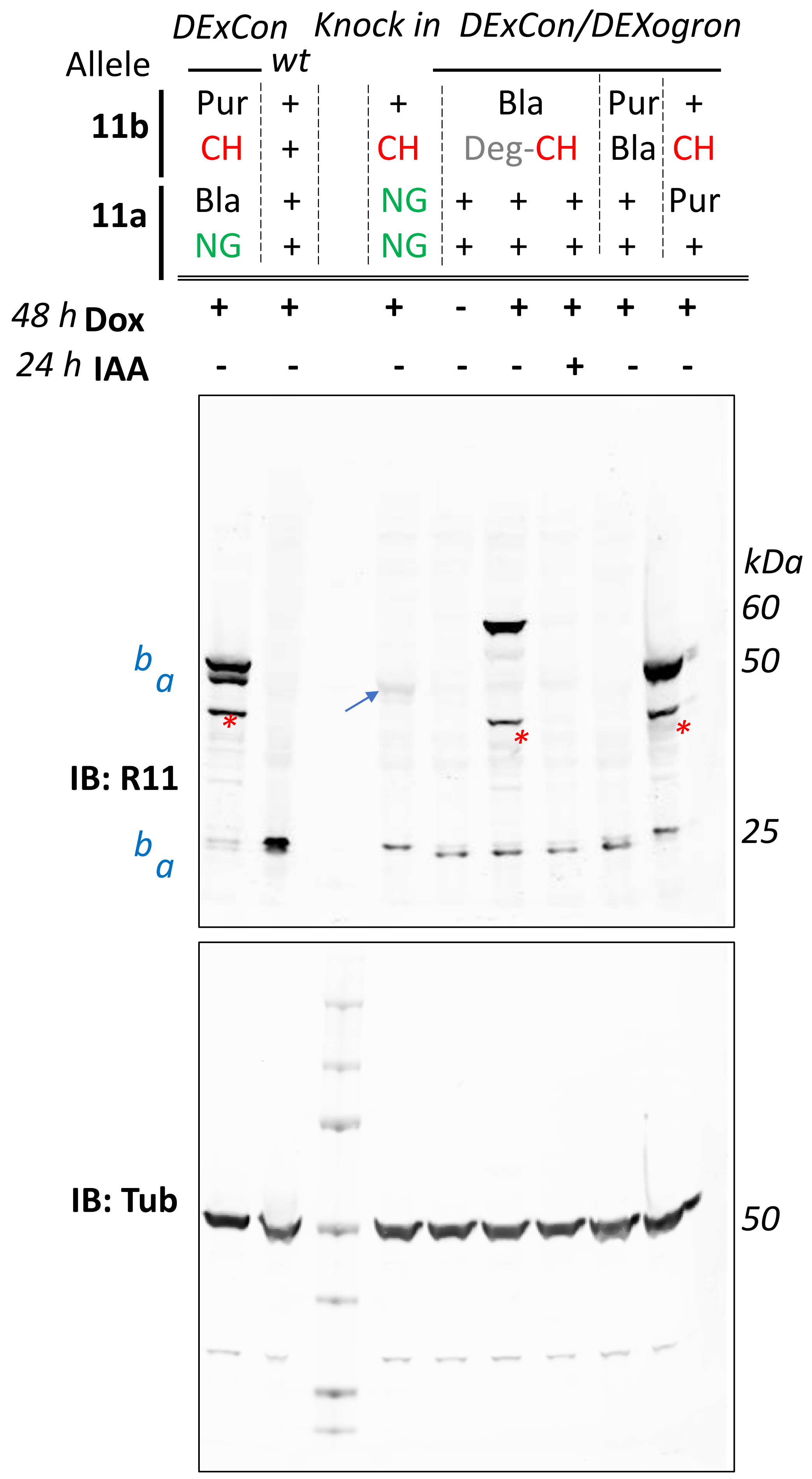

Figure 6-figure supplement 1 - source data 5

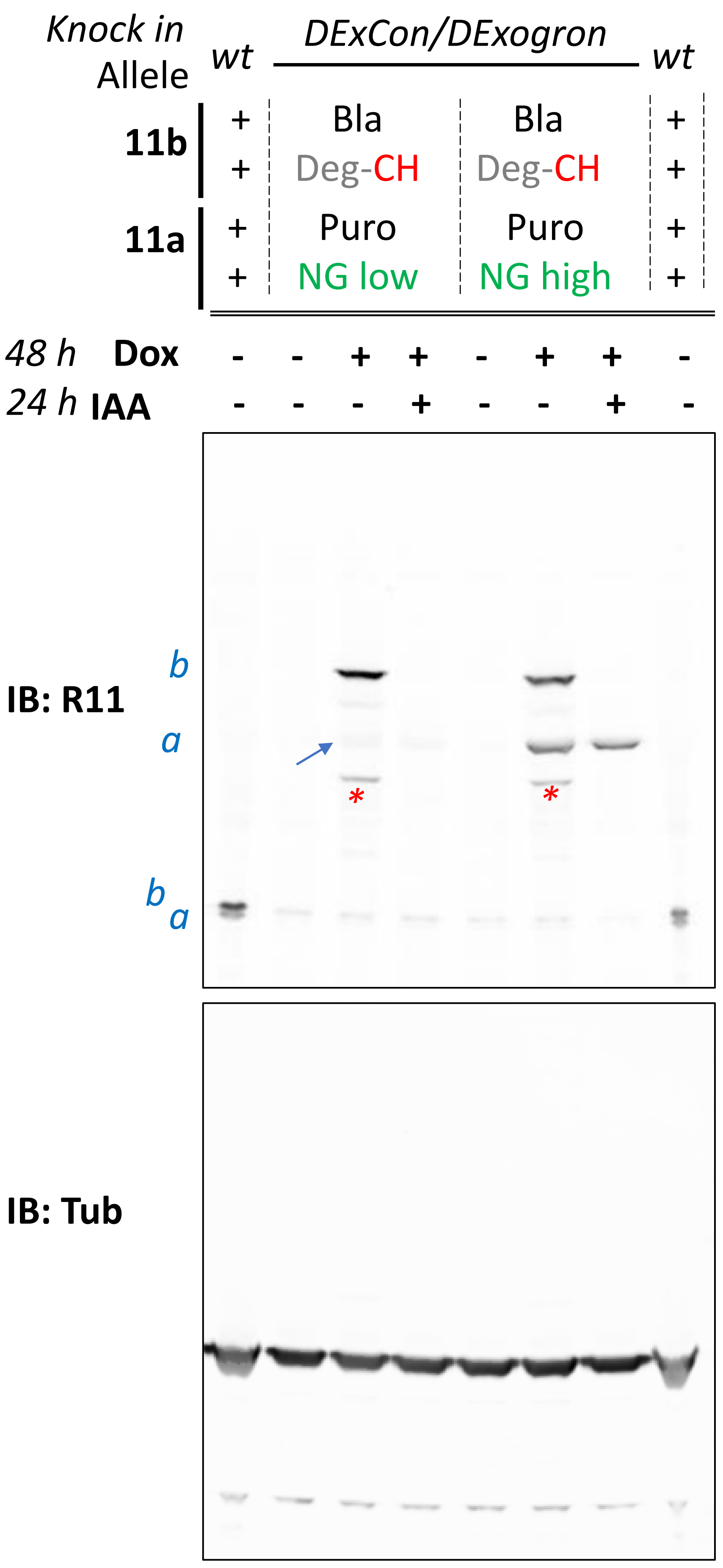

Figure 6-figure supplement 2 - source data 1

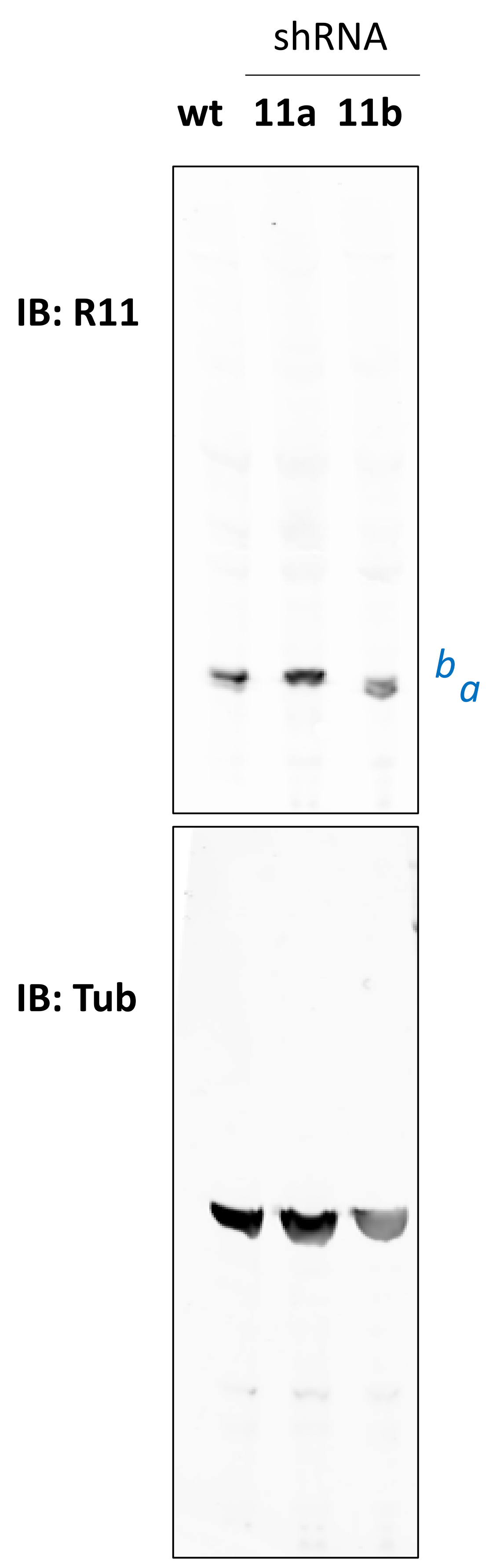

Figure 6-figure supplement 4 - source data 1

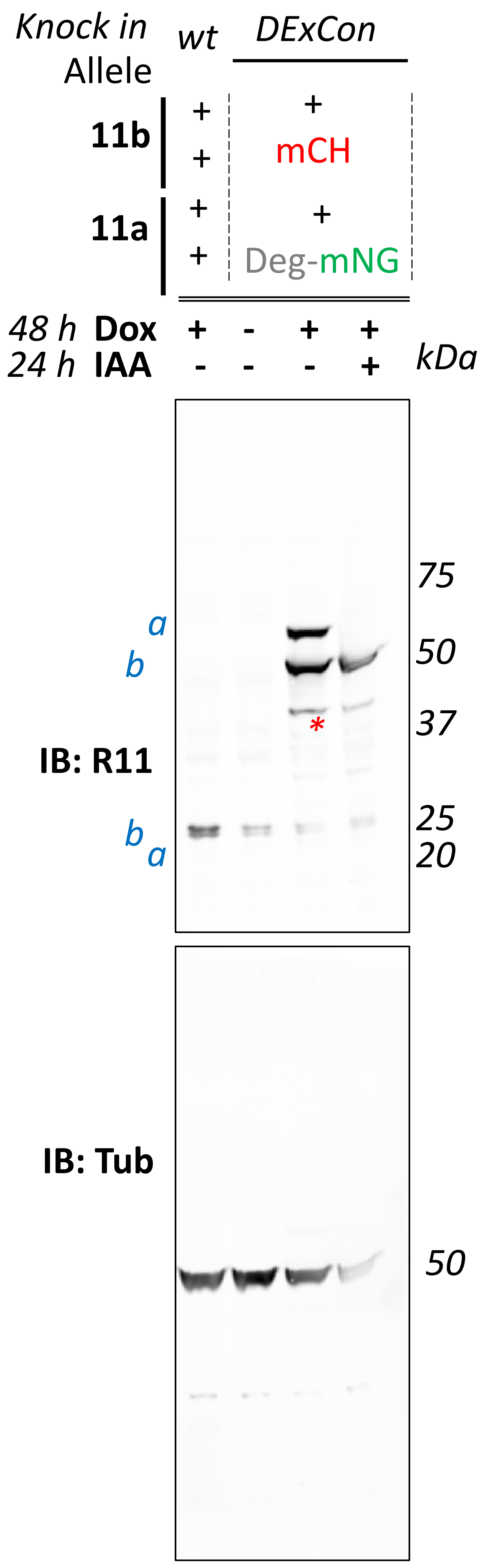

Supplement: Source data 1. — Annotated uncropped blots for Figures 1—3, Figure 5, Figure 6, Figure 1—figure supplement 2, Figure 2—figure supplement 1, Figure 3—figure supplement 1, Figure 4—figure supplement 1, Figure 6—figure supplements 1 and 2, Figure 6—figure supplement 4. [file elife-76651-data1.pdf]
